# Paraffin Graphite Composite Spheres for Thermal Energy Management

**DOI:** 10.3390/ma18071482

**Published:** 2025-03-26

**Authors:** Gyorgy Thalmaier, Nicoleta Cobîrzan, Niculina A. Sechel, Ioan Vida-Simiti

**Affiliations:** 1 Faculty of Materials and Environmental Engineering, Technical University of Cluj-Napoca, 103 Muncii blv., 400641 Cluj-Napoca, Romania; niculina.sechel@stm.utcluj.ro (N.A.S.); vida.simiti@stm.utcluj.ro (I.V.-S.); 2 Faculty of Civil Engineering, Technical University of Cluj-Napoca, 15 C. Daicoviciu, 400020 Cluj-Napoca, Romania; nicoleta.cobarzan@ccm.utcluj.ro; 3 Technical Science Academy of Romania, Dacia Ave., 26, 010413 Bucharest, Romania

**Keywords:** 3D printing, fused deposition modeling, PCM composite, injection molding

## Abstract

The paper presents a simple and cost-effective way of enhancing the thermal conductivity of the paraffin/graphite phase change material (PCM) composite spheres manufactured by using a low-cost and eco-friendly method. The composite materials were made of an admixture of 5–20% vol. graphite powder. The manufacturing process of macro-encapsulated PCM consists of creating digital models, mold printing, and PCM injections. The experimental data shows that composite materials have an increased thermal conductivity, from 3 to 11 times compared to paraffin, and are effective in cooling application of electronic components where they lowered the maximum temperature up to 30 °C. For low-volume PCM sphere fabrication, it was proposed the injection molding in the 3D printed mold; the results show that fused deposition modeling (FDM) is efficient in saving energy up to 30% compared to machining. The carbon emissions generated during the fabrication technology were found to be strongly dependent on printing process parameters and the energy mix used to produce the electrical energy used.

## 1. Introduction

Phase change materials (or latent heat storage materials) are substances that undergo physical transformations and provide latent heat at constant temperatures. These transformations take place due to PCM composite melting/solidification [1]. Materials with high latent heat capacity of melting/solidification can be used as an efficient method to store thermal energy [2]. Thus, the energy per unit mass is absorbed and stored during the phase change (melting) and released when the external temperature drops below the solidification temperature.

The use of phase change materials for thermal energy storage provides an adequate and cheap solution that improves the performance of energy systems [2] and, therefore, reduces the demand for non-renewable energy.

PCM has a high potential to be used for heat temperature control in buildings [3,4], automobiles [5], agriculture [6,7,8], electronics [9], and other fields.

Technology and innovation in the field of electronics had a dynamic evolution, gradually becoming more performant, with higher working speed and smaller dimensions. Their increased performance has led to the generation of a high temperature that can affect efficiency and lifespan when the temperature exceeds the safety limits [10,11]. Phase change materials represent an efficient solution in temperature control, keeping the temperature below the critical point [12]. The use of PCM in the field of electronics has been studied by several authors, highlighting their efficiency and benefits in relation to the materials used [12,13,14]. Zhao et al. [15] developed and tested a new composite material made of paraffin wax, graphite, and metal alloy for application in passive cooling cell and battery modules. The graphite foam-based composite PCM was studied by Liu et al. [16], highlighting the impact on heat transfer of cavities, structure, and tilt angles. Wang et al. [17] numerically investigate a new HS-PCM module studying the influence of copper plate with no PCM contact in the thermal control of electronic devices.

Paraffin, as PCM, are saturated hydrocarbons with the general formula C_n_H_2n+2_, which have a high latent heat capacity [18] and a variable phase change temperature. They are non-toxic materials, have a low cost, and have a good energy storage density. The phase change temperature of PCM increases with the carbon chain’s length and can be at room temperature in a solid or liquid state. Their performance is dependent on composite thermal conductivity, which may be increased by introducing a metallic foam [19,20] type skeleton or by dispersing in their mass the metal particles with high thermal conductivity [21,22]. The latter, although it can be a simpler way of production, is prone during the phase change transitions to sedimentation in the liquid state of the added particles.

The PCM materials are used in an encapsulated form to prevent PCM leakage and its degradation caused by the environment. Depending on their size, the encapsulated PCM are of 3 types: nanoencapsulated with size < than 1 µm, microencapsulated with sizes between 1–1000 µm, and macroencapsulated when their size is over 1 mm. The micro and nano-encapsulation offer clear advantages in terms of volume-to-surface ratio, making the PCM react faster. However, the encapsulation materials volume fraction can be up to 50%, lowering the energy storage density [23].

Due to their high energy density, macroencapsulated PCMs have raised attention in areas like energy-efficient buildings or storing solar energy [24]. Most commonly, macroencapsulation is done by manufacturing PCM pouches, tubes filled with the PCM, or PCM spheres. Most capsules are rigid and do not allow the deformation of the material, even in the case of PCMs with high volume variation during the melting.

Shape-remodeled PCM macrocapsules were developed by D.-H. Yu [24], in which the flexible shell allows the macrocapsule to remodel its shape repeatedly when needed and to expand. From the user’s perspective, spherical PCMs are largely preferred since, in this way, the energy density and versatility are maximized, being easier to use and produce.

The most efficient way for the industrial manufacturing of this type of part (PCM spheres, but not limited to this shape) is the injection molding process due to the use of multi-cavity molds and high-volume capabilities.

Fused deposition modeling (FDM) is a simple way of manufacturing complex shapes and is affordable. This 3D printing technique mainly uses inexpensive polylactic acid (PLA) to fabricate the needed parts. The material parameters of these filaments are high enough to be able to withstand the stresses implied by the injection molding of the PCM spheres.

3D-printed molds are not a new concept in injection molding, but they are a revolutionary way to create mold prototypes. In the right circumstances, they give a different perspective, allowing the 3D printing process to manufacture industrial-level molds and help expand opportunities in the industry.

This paper proposes a new paraffin composite material manufactured by using a facile and eco-friendly technology. The process consists of creating the digital model (a), mold printing (b), and PCM injection (c). The resulting manufactured spheres can be integrated into manufacturing shape-remodeled PCM macrocapsules [24] as a cheap, affordable, and simple way of producing a composite for the passive cooling of electronic devices. The present study evaluates the characteristics of PCM composites and the molding environmental impact, proposed to sustain the long-term efficiency and reliability of electronic components.

## 2. Materials and Methods

### 2.1. Paraffin Composite Fabrication and Characterization

**Figure 1:**
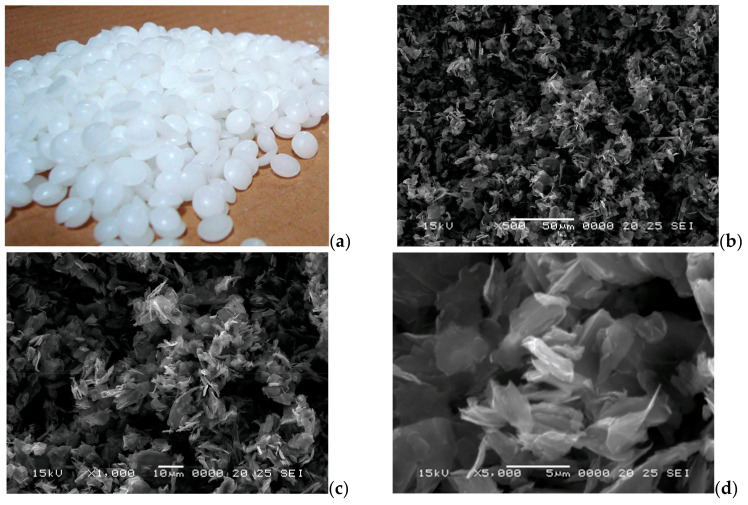
The used starting materials: paraffin (**a**) and graphite powder at different magnifications (**b**) 500×; (**c**) 1000×; (**d**) 5000×.

The paraffin composite material was produced by using a technical purity paraffin (C_n_H_2n+2_, n = 25–30) with a melting range of 51–57 °C and a latent heat of fusion of 170 kJ/kg and an excellent graphite flake (~0.15 µm thick, <5 µm in length Figure 1b). The graphite powder was added in different percentages (5, 10, 15, 20% vol) as a thermal conductivity enhancer. The 20% limit was imposed for the composite to maintain a high energy absorption capacity since the added graphite does not have a phase change capacity. The size and shape of the graphite powder were chosen to minimize its sedimentation behavior in the composite (The sedimentation speed calculated according to Stokes law [25] is <0.15 mm/h).

In the first stage, the graphite powder was introduced into the heated liquid paraffin at 72 °C. Homogenization of the samples was achieved through continuous mixing for about 5 min until the graphite particles were in suspension and had a homogeneous appearance, according to the melt blending process. The obtained mixture was used in the present form or after reheating for the specimen’s manufacture and thermal conductivity measurements.

The thermal conductivity of the composites was measured on an experimental setup presented elsewhere [26], having a 3 cm basalt wool insulation with a thermal conductivity of 0.035 W/mK. The thermal conductivity measurements were done in the 25–35 °C range. The melting and freezing behavior of the samples was evaluated using differential scanning calorimetry (DSC) in the air using a heating rate of 1.5 °C/min.

Specific heat (Cp) of paraffin composites was estimated using the rule of mixtures. The thermal diffusivity (α) was calculated by dividing the thermal conductivity (λ) by the product of the density (ρ) and the specific heat capacity C_p_ (α = λ/C_p_·ρ).

### 2.2. Three D Printing of the Mold

**Figure 2:**
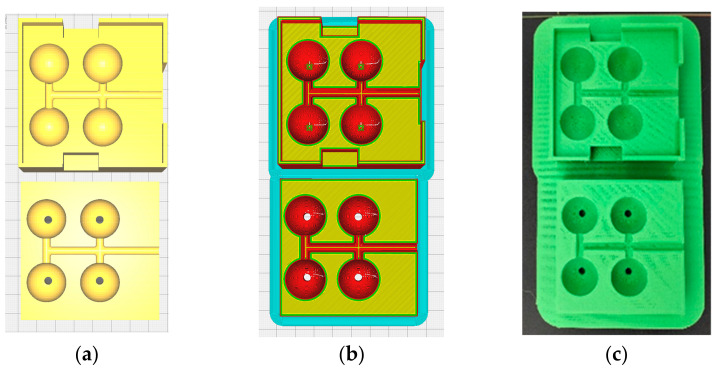
The mold in the 3 stages (**a**) 3D drawing, (**b**) after slicing, (**c**) after print.

The mold was manufactured from a standard PLA filament using FDM printing technology (Figure 2). The digital model of the mold was created using the SolidWorks 2022 environment.

The slicing and print parameter control was done in the Cura software environment (version 4.13.1), and the mold was printed on a Creality Ender 5 pro FDM printer (Shenzhen, China). The main printing parameters were characteristic for the polylactic acid (PLA) filament (print temperature 210 °C, bed temperature 60 °C) and infill 100%. The environmental impact of mold manufacturing is highly influenced by the 3D printer and the printing parameters. Therefore, the CO_2_ emissions generated by the filament during its fabrication process and the electric energy consumed for the printing of the parts are also considered in the environmental assessment. For simplicity, to calculate the CO_2_ generated, it was used the conversion factor for electric energy [27].

### 2.3. Injection Moulding of the Composite

The injection process involves feeding the mold with the composite in a liquid state through the feeding channel of the mold. Before injecting the samples, the mold was heated to 70 °C at a constant temperature water bath, maintained for 10 min to prevent premature solidification and to ensure a complete filling of the cavities. The mold was previously coated with grease to facilitate the extraction of the spheres. After that, the mold reached a temperature of 70 °C, and the molten composite held at 70 °C was also injected into the mold. The whole system was left to cool to room temperature, and after reaching the temperature of 35 °C, the spheres were extracted from the mold.

### 2.4. Assessment of Paraffin Composite for Passive Cooling of Electronic Devices

This experiment involves testing the paraffin composite in simulated conditions for application in an electronic component backup cooling system. For this experiment, a 75 Ω resistor was used and connected to a direct current (DC current) source that would heat the resistor by applying a voltage of 15 V and a current of 0.7 A. The dissipated power was 10.5 W. The resistor was placed in a reservoir with sufficient paraffin composite (~75 mL), and its temperature was monitored by a thermocouple connected to a data acquisition system. The sampling interval was 60 s, and the total duration for each experiment was 25 min. The temperature reduction of the resistor was considered the temperature difference between the maximum temperature reached in the air and the maximum temperature reached using the different cooling systems.

## 3. Results

### 3.1. Thermal Characterization of the Paraffin Composite

#### 3.1.1. Differential Scanning Calorimetry (DSC)

**Figure 3:**
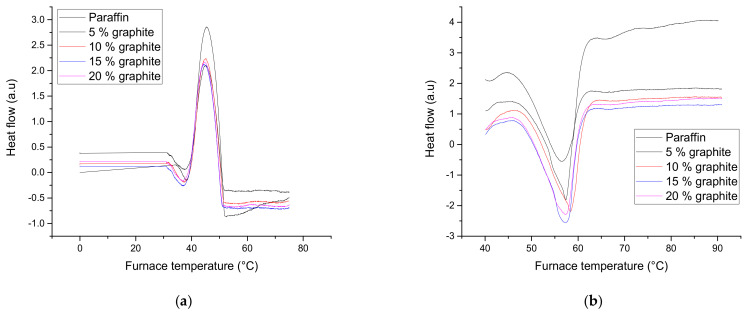
DSC curves for the samples studied: (**a**) heating; (**b**) cooling.

By performing differential scanning calorimetry, the phase change (melting or solidification) of the four PCM composites is highlighted (Figure 3). The DSC measurements were performed up to 100 °C in the air using a heating and cooling rate of 1.5 °C per minute and holding at the maximum temperature for 60 s.

In the DSC curves resulting from the measurements on the 4 sample types, two peaks are present, one endothermic (melting) and one exothermic (solidification)—Figure 3. The temperature evolution over time is indicated by the black curve. As can be observed, the melting of paraffin composite starts at a temperature of around 46 °C and ends at 62 °C. The peak positions are around 57 °C. The composite solidification starts at 51 °C and ends at 38.5 °C. The peak positions at solidification are located around 45 °C. No variation of critical points with the carbon content was observed.

Temperature differences were observed at melting and solidification due to thermal hysteresis. There are no differences in the peak area (the samples had similar mass), and the amount of assimilated or yielded heat decreases with the increase in the amount of added graphite. This decrease is because carbon does not participate in the phase change.

#### 3.1.2. Thermal Parameters

The thermal conductivity was determined by using a steady-state heat method. This method involves the use of standard samples in a standard-sample-standard system. Temperatures are measured with platinum-rhodium thermocouples.

Thermal parameters of paraffin composite are presented in Table 1.

When 5%, 10%, 15%, and 20% graphite powder were added into paraffin, an increase of 3 to 11 times in thermal conductivity and thermal diffusivity was obtained in comparison to paraffin only. Also, a decrease of up to 1% for specific heat was obtained for a paraffin composite with a content of 20% graphite powder.

Improvements in the thermal conductivity of paraffin with nanographene were obtained by Ali et al. [28], of graphite and boron nitride by Chen et al. [29], of graphene/paraffin nanocomposite by Joseph and Sajith [14], and of paraffin with copper foam by Zhou and Qiao [30].

In their research, Ali et al. [28] combined paraffin with nanographene in a content of 1%, 2%, and 3%, obtaining an improvement in thermal conductivity of 1.43, 1.73, and 2.46 times compared to that of the PCM. Chen et al. [29] combined paraffin with graphite and boron nitride at max percentages of 40% wt. Following the measurements, a composite material with thermal conductivity from 0.24 to 1.3 W/m K was obtained.

Joseph and Sajith [14], in their experimental research on the paraffin/graphene composite proposed for thermal control in electronics, obtained an improvement of thermal conductivity up to 59.5% for a content of 0.5 wt% graphene. Zhou and Qiao [30] combined paraffin with copper foam, showing an important improvement in thermal conductivity up to 12 w/Mk.

Comparing the results obtained by the authors [14,28,29,30] with the results obtained in this research study, the same trends in improvement of the thermal conductivity of paraffin composite may be seen.

### 3.2. Characterizantion of Composite Tested in Simulated Conditions

This experimental research involves testing the material in simulated conditions for the assessment of its potential for cooling off electronic components. The most used cooling method for electronic components is the usage of aluminum or copper heat exchangers; their radiating surface area can be increased using fins. During experiments, the aluminum heat exchanger was compared with the effect of the manufactured phase change composite.

**Figure 4:**
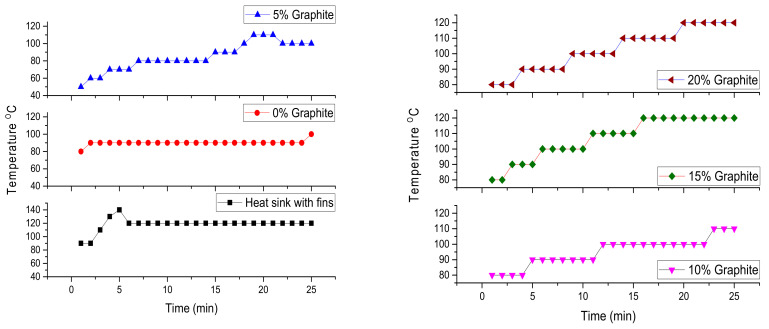
Temperature evolution in passive cooling experiment with different cooling strategies.

A significant difference can be seen between the temperature recorded on the electrical resistance cooled by the aluminum heat exchanger and that embedded in the pure phase change material (Figure 4). The temperature differences between the two curves (~30 °C) are due to the cooling effect by the PCM (paraffin), which captures a good part of the thermal energy from the resistors’ surface.

Of the four PCM composites with 5%, 10%, 15%, and 20% graphite content, it can be observed that the temperature has a slight tendency to increase with the increase of graphite content in the composite. This increase in temperature is due to decreases in the heat capacity of the phase change material. By adding graphite powder to PCM, its thermal conductivity was considerably improved, but its ability to store thermal energy decreased (Table 1).

### 3.3. Prototype Sphere Fabrication by Injection in the Molding of the Paraffin Composite

#### 3.3.1. 3D Printing of the Mold

During the drawing phase, multiple nest injection molding was chosen to increase the productivity of the injection process. The mold contains four identical spherical nests with the corresponding channels for demonstration purposes. The mold was designed with two halves to facilitate the spheres’ extraction after the injection. The mold was created by FDM by stepwise deposition of a thin strip (0.4 mm) of PLA until the whole parts were created on a heated bed that descends on every layer, with the layer height, which in this case was 0.2 mm.

The eco-friendly nature of the manufacturing process is given by its nature since it can reduce energy consumption by using an optimized fabrication technology [31]. Additionally, the choice of the material, since the polylactic acid is made from renewable material—cornstarch [32], being a recyclable and biodegradable material increases, further the sustainability of this process. Using 3D printing, the specific bottom-up approach minimizes the generated waste and further increases the eco-friendly nature of the manufacturing process [33]. Of the 175 g PLA used for the printing, less than 3 g (~1.7%) was technological waste under the form of the skirt used and the initial filament extrusion.

#### 3.3.2. Energy and Eco-Impact Evaluation of 3D Printing of the Mold

**Figure 5:**
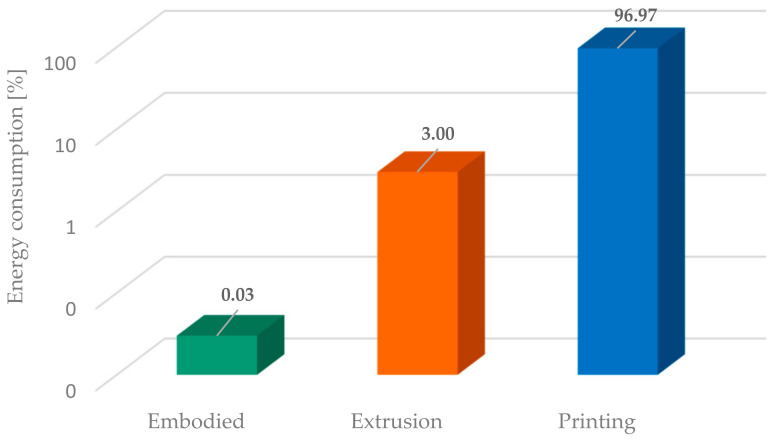
Energy consumption percentage at the phases of the FDM process.

The energy consumed for the printing process of the parts (with the cumulated volume of 136 cm^3^) was measured at 1.8 kWh. From this energy, approximately 7% was consumed during the printer nozzle and bed heating, while the rest was consumed during the printing process. The embodied energy of the product resulted in the sum of energies consumed for the extrusion of the filament (0.322 kWh/kg [34]), the 3D printing (10.6 kWh/kg), and the production of paraffin composite/mold (0.0033 kWh/kg [34]). Adding all these values results in a total embodied energy of 10.9 kWh/kg (Figure 5).

**Figure 6:**
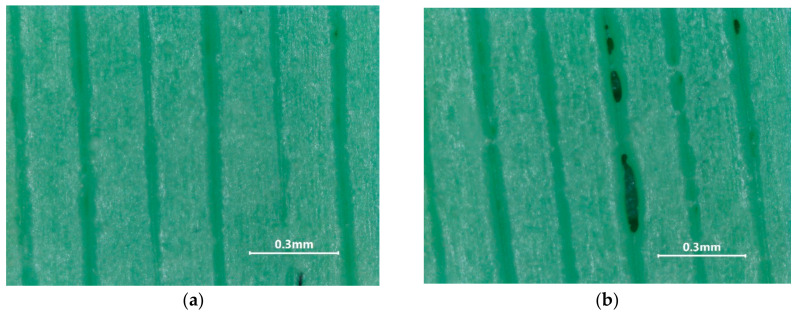
Defect densities in the printed half-molds: (**a**) infill 100%, (**b**) infill 50%.

As presented in Figure 5, by far, the most energy-intensive step is the printing process, which needs to be optimized to decrease energy consumption. By reducing the infill to 50%, the energy required for printing may be reduced to 9.3 kWh/kg (from 10.6 kWh/kg), a reduction of approx. 15%. However, the defect densities (void) in the printed part were significantly increased as presented in Figure 6. The generated voids permit the liquid PCM to infiltrate the mold, complicating the extraction process.

**Figure 7:**
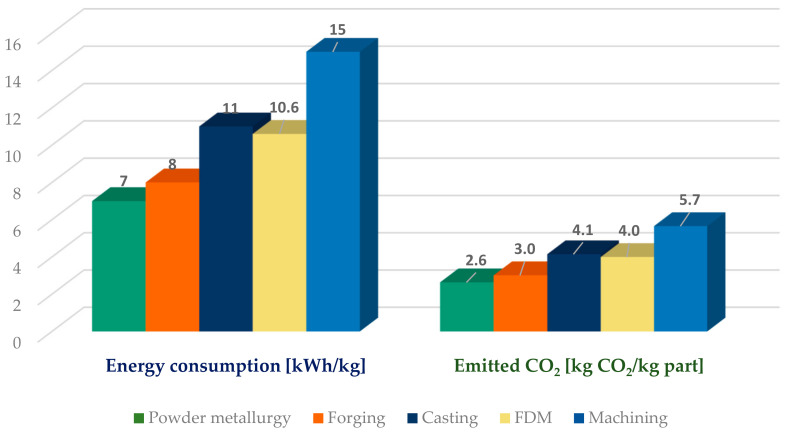
Comparison of median energy consumption and CO_2_ generated by using different manufacturing technologies.

The carbon emissions generated by the printing process are strongly dependent on the energy mix present locally. The greener energy is included in the mix, the lower the carbon emissions will be. Europewide, the EU Commission regularly presents these coefficients for each member state [27]. The values are between 0.012 for Norway and 0.776 for Poland, corresponding to the last published year, 2021. Romania’s energy mix contains a good portion of hydro and solar energy, so the coefficient is 0.377. The 10.9 kWh of energy used for printing corresponds to the emission of 4.1 kg of CO_2_ into the atmosphere (Figure 7). As a comparison, Enemuoh et al. [34] measured energy consumption for plastic injection molding of PLA parts using a single nest mold to be 36.49 kWh/kg, corresponding to 13.8 kg of emitted CO_2_.

Conventional manufacturing technologies were optimized throughout the year, are extremely efficient from all points of view, and are high-volume manufacturing processes. Compared to them FDM manufacturing is far from being considered optimized and is a low-volume manufacturing process. All presented processes in Figure 7 use electricity to manufacture; the values for them are from [35], except in the case of FDM, where the values were measured and calculated.

Powder metallurgy is considered a green manufacturing process, so it is not a surprise that the energy used and the emitted CO_2_ are lower in comparison to conventional manufacturing processes. The highest energy consumption seems to be in the case of machining. Although it is a new technology, the FDM process has a medium energy demand, being close to the casting process.

Due to the tendency for continuous improvement in the FDM process, it is expected that energy consumption will be further reduced over time. Also, the tendency to include green energy sources in the power grid production will reduce further CO_2_ emissions. Considering Norway’s energy grid, which uses electrical energy mix, we may assume that carbon emissions may be reduced by approx. 97%.

#### 3.3.3. Injection of the Paraffin Composite Inside the Molding

The paraffin composite was heated to 70 °C in a constant-temperature water bath, maintained for 10 min to ensure a uniform temperature and water-like viscosity. The melted composite was transferred to a 50 mL syringe, which was used to hand-fill the mold.

**Figure 8:**
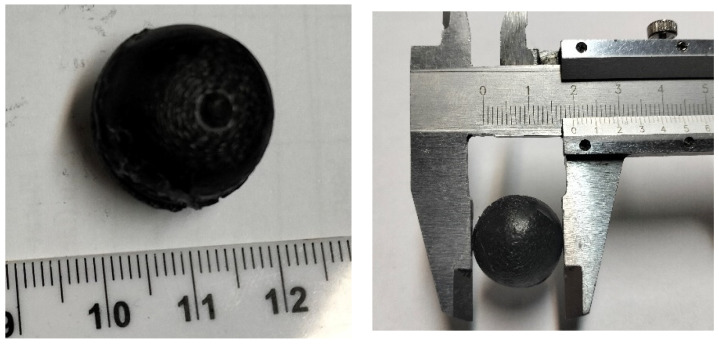
Paraffin composite spheres made by injection.

The mold was considered filled when the liquid composite started leaking through the top vent holes. This also meant that the coated silicone grease between the bases successfully prevented the leakage of liquid PCM and closed the printing defects, too. After solidification, the mold was opened, obtaining the paraffin composite spheres (Figure 8).

**Figure 9:**
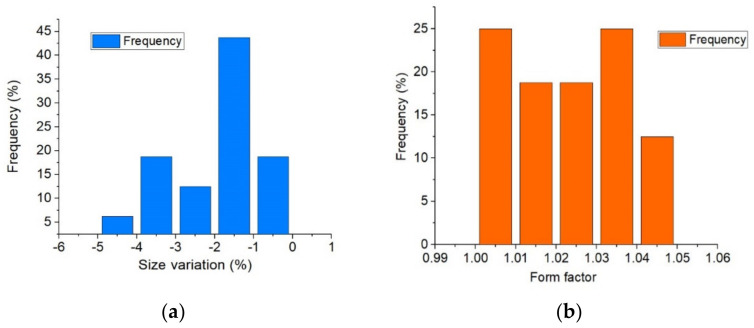
Size variation (**a**) and form factor distribution (**b**) of the manufactured spheres.

As shown in Figure 8, the paraffin composite spheres had a median diameter of 19.7 mm. The surface correction with silicon grease inside the nests of the mold produced a dimensional reduction of about 1.5%. The obtained spheres had a high circularity, presented a form factor close to unity (see Figure 9), and had a coefficient variation of 0.01361.

## 4. Conclusions

In the present work, a new paraffin composite material with a spherical shape was manufactured using an eco-friendly technology with a lower environmental impact than other conventional fabrication technologies.

High-quality paraffin-graphite composites were fabricated using the melt blending process. These composites begin to melt around 46 °C, and this process ends at 62 °C. As expected, no variation of critical points with the added carbon was observed. The composites assimilated or yielded heat decreased with the increase in the amount of added graphite.

A strong increase in thermal conductivity can be observed up to 11 times compared to the thermal conductivity of the paraffin when 20% graphite was added. Due to its high thermal conductivity and PCM characteristics, this material can be used for different component cooling applications. During the laboratory tests, the maximum temperature difference recorded on the electrical resistance cooled by the aluminum heat exchanger and that embedded in the pure phase change composites was ~30 °C.

This cooling process can be considered a green process since it does not generate supplementary CO_2_. This idea was further assured by the proposed technology for mold fabrication to manufacture paraffin spheres in low volume, namely FDM, using PLA.

Although it is a new technology, the FDM process has a medium energy demand, being close to the casting process. Due to the tendency of continuous improvement in the FDM process, it is expected that the energy consumption to be further reduced over time. Also, the tendency to utilize more green energy sources in the power grid will also reduce CO_2_ emissions.

## Figures and Tables

**Table 1:** Thermal properties of paraffin composite as a function of the graphite content.

Graphite Content (vol. %)	0	5	10	15	20
Thermal conductivity (W/mK)	0.25	0.74	0.88	1.15	2.72
Specific heat (J/kgK)	2144	2114	2082	2048	2012
Thermal diffusivity (m^2^/s)	1.3	3.9	4.6	5.9	13.7
Density (kg/m^3^)	897	925	955	987	1020

## Data Availability

The original contributions presented in this study are included in the article. Further inquiries can be directed at the corresponding author.
